# PALLIATIVE CARE NEEDS ASSESSMENT OF A RURAL AND FRONTIER STATE

**DOI:** 10.1093/geroni/igz038.486

**Published:** 2019-11-08

**Authors:** Catherine Carrico, Andrea Slosser, Robin A Barry, Christine McKibbin

**Affiliations:** University of Wyoming, Laramie, Wyoming, United States

## Abstract

Background: Palliative Care (PC) is a valuable tool for improving the lives of people living with chronic illness. However, access to PC is limited in rural areas. The purpose of this study is to describe the current PC needs and barriers to care in a rural state. Methods: An online survey was disseminated through professional organizations, licensing boards, and the University of Wyoming. Rurality of counties was classified using Rural Urban Continuum Codes (RUCC). Descriptive statistics were calculated using SPSS. Results: Responses were received from 336 individuals across 20 of 23 counties (i.e., RUCC range 3 - 9; 1=most metropolitan, 9=most rural). The majority worked in healthcare or social service sectors (n = 265, 78.8%). Approximately one-half (n = 119, 50.6%) of these individuals endorsed providing PC (i.e., typically symptom management, supportive resources, and family support). Over one-half of respondents (n = 173, 51.5%) rated availability of PC services in their communities as “poor” or “somewhat good.” Key barriers to providing PC were lack of patient information and knowledge (n = 215), access to PC specialists (n = 183), and funding and reimbursement (n = 181). Approximately one third of healthcare professionals (n = 78, 32.8%) had received formal training in PC. A majority of healthcare and social service respondents (n = 139, 59.1%) endorsed interest in PC continuing education. Conclusions: This study provides insight into the state of PC across a rural state. Results highlight the need to design accessible education and implement system transformation to improve PC access.

